# A common genomic code for chromatin architecture and recombination landscape

**DOI:** 10.1371/journal.pone.0213278

**Published:** 2019-03-13

**Authors:** Kamel Jabbari, Johannes Wirtz, Martina Rauscher, Thomas Wiehe

**Affiliations:** Institute for Genetics, Biocenter Cologne, University of Cologne, Köln, Germany; Tulane University Health Sciences Center, UNITED STATES

## Abstract

Recent findings established a link between DNA sequence composition and interphase chromatin architecture and explained the evolutionary conservation of TADs (Topologically Associated Domains) and LADs (Lamina Associated Domains) in mammals. This prompted us to analyse conformation capture and recombination rate data to study the relationship between chromatin architecture and recombination landscape of human and mouse genomes. The results reveal that: (1) low recombination domains and blocks of elevated linkage disequilibrium tend to coincide with TADs and isochores, indicating co-evolving regulatory elements and genes in insulated neighbourhoods; (2) double strand break (DSB) and recombination frequencies increase in the short loops of GC-rich TADs, whereas recombination cold spots are typical of LADs and (3) the binding and loading of proteins, which are critical for DSB and meiotic recombination (SPO11, DMC1, H3K4me3 and PRMD9) are higher in GC-rich TADs. One explanation for these observations is that the occurrence of DSB and recombination in meiotic cells are associated with compositional and epigenetic features (genomic code) that influence DNA stiffness/flexibility and appear to be similar to those guiding the chromatin architecture in the interphase nucleus of pre-leptotene cells.

## Introduction

More than three decades ago, a relationship between genome organization and recombination was detected from banding of human chromosomes. R bands and G/R borders were found to be the preferred sites of DNA exchanges and the “hot spots” of mitotic chiasmata [1,2]. These observations suggested a correlation of recombination with compositional discontinuities (change of GC% along chromosomes) [3–5], which was later extended and statistically validated [6].

At a broad scale, it was shown that recombination is favoured or suppressed in specific mega-base sized regions [7] and that linkage disequilibrium (LD) and GC% are interdependent, with strong LD being typical of GC-poor and less genetically diverse genomic regions [8,9]. Although recombination is known to be mutagenic and biased [10–13], whether the correlation between GC level and recombination is driven by GC-biased gene conversion [14–17] or whether local GC content itself is driving recombination [18–24], is still a matter of debate. Yet, in human, hotspots of recombination are associated with local GC spikes (1 to 2 kb in size) and with an increase of the local mutation rate resulting in G or C nucleotides, but without an effect on substitution rate or divergence [21]. This is consistent with the observation that while local structure of recombination is fast-evolving [22,23], rates over broad scales appear to be constrained [24]. These observations are in line with early proposals that meiotic proteins loading on chromosomes is modulated by isochores and that R-bands differentially favor the formation of double-strand breaks (DSBs) during meiosis [25,26], thought to be primarily determined by the chromatin fibre stiffness [26].

In addition to the occurrence of DSBs, the entry of pre-leptotene spermatocytes into meiosis is accompanied by several epigenetic changes. In primates, as well as in mice, the location of most DSBs is correlated with the trimethylation of histone 3 lysine 4 (H3K4me3) by the DNA binding enzyme PRDM9 [27–31]. Interestingly, PRDM9 is absent in dogs [32] and truncated or absent in several fish and bird species, suggesting that PRDM9 does not direct recombination events in these species, where meiotic recombination tends to occur near promoter-like features [33–35].

In the present work we show results of a large-scale analysis of chromatin folding and recombination hot-spots by aligning chromatin interaction maps with recombination frequency profiles and LD-blocks. Focusing on the regional scale of Topologically Associated Domains (TADs) [36,37] and Lamina-Associated Domains (LADs) [38], we noticed a non-random association of chromosomal recombination profile and chromatin architecture. TADs represent an assembly of chromatin loops (with boundaries of 0.2–2 Mb in size [36,38–40], which can be resolved into contact domains, of 185 Kb in median size [39]. LADs have a median size of 500 Kb, are AT-rich and distributed over the whole genome [38,40, 41]. To ascertain the co-mapping of recombination landscape and TADs, we studied the genomic distribution of DSBs, and binding/occupancy sites of recombination machinery components. We argue that these properties together with the correspondence between LD-blocks and TADs hint to the existence of a common “genomic code” of chromatin architecture in meiotic and mitotic cells.

## Material and methods

### Recombination and LD-blocks data sets

Multiparent populations such as the Diversity Outbred (DO) mouse stock represent a valuable resource of large numbers of crossover events accumulated in present day siblings from founder haplotypes. To establish chromosome profiles of recombination activity in mouse, we made use of coordinates and haplotypes of distinct (not identical by descent) crossovers (COs) and locations of reduced recombination domains obtained from [34]. This study provides a high-density genotype data set from 6886 DO mice spanning 16 breeding generations, in which 2.2 million CO events were localized in intervals with a median size of 28 kb. By filtering out COs identical by descent, the authors obtained a set of 749,560 distinct COs and used a measure called “CO information score” to calculate deviation from the expected frequency of COs with respect to founder strain pairs; this score takes larger values when recombination rate is low.

For the analysis of human data, we used linkage disequilibrium in human populations. LD is a measure of alleles co-inheritance expressed in terms of the squared correlation (r^2^) between alleles at a pair of single nucleotide polymorphism (SNP) loci (*e*.*g*., [42]). We used the LD-blocks determined in [43] for populations of European (CEU, TSI, GBR, FIN and IBS), African (YRI, LWK and ASW) and East Asian (CHB, JPT and CHS) descent from the 1k human genomes project phase I [44]. This data set comprises 2605, 1467 and 1725 LD-blocks in African, East Asian and European populations, respectively. To have an overall view on recombination activity across the human genome, we used the human sex-averaged recombination map available at the ftp site of the 1k genomes project (HapmapII_GRCh37_RecombinationHotspots.tar.gz).

### Isochores and TAD data sets

We obtained TAD boundaries identified by Hi-C experiments on human and mouse embryonic stem cells from [36]. The UCSC batch coordinate conversion (liftOver at http://genome.ucsc.edu/cgibin/hgLiftOver) was used here to convert human (hg18 to hg19 assembly) and mouse (mm9 to mm10 assembly) isochore coordinates reported in [45,46]. Isochore maps were visualized with "draw-chromosome-gc.pl" [47]. Hi-C maps were drawn using Juicebox [48] and the 3D Genome Browser for comparative Hi-C plots [49]. For crosschecking purpose, we also used the recently published high resolution Hi-C data from mouse sperm [50], GEO accession: GSE79230 and mouse neural embryonic stem cells [51], GEO accession: GSE96107.

### ChIP-Seq and sequencing data

To further validate and study in more detail the relationship between recombination and chromatin architecture, we analysed other markers and events known to be crucial for recombination activity. Meiotic DSBs are induced by dimers of the conserved topoisomerase-like protein SPO11 and release of covalently bound SPO11 short oligonucleotides (SPO11-oligos) [52]. By sequencing DNA fragments that remain attached to SPO11 and mapping SPO11-oligo reads [53] of mouse spermatocytes, a total of 13.960 DSB hotspots were defined [54]. We used these data to study the distribution of DSBs along chromosomes. The genomic coordinates of binding or occupancy sites of recombination machinery components, namely, DMC1, H3K4me3 and the DNA-binding sites of the zinc finger, histone methyltransferase PRDM9 were obtained from [54, 55].

### Statistical analysis

We asked whether TADs, isochores and LD-blocks overlap more than expected by chance. To this end, we performed an association analysis of genomic regions [56] based on permutation tests using the R/Bioconductor package *regioneR* [57], as in [41]. This method is graphically summarized in Figure A in S1 File. The permutation test is supported by the *permTest* function of *regioneR*, it evaluates the non-random overlap between two sets of genomic intervals, low *p*-values indicate non-random association between the two sets under consideration. When performing an association analysis, it is possible to detect associations that do not reflect boundary proximity (e.g. TADs *vs*. LD-blocks boundaries). To test for boundaries match between two sets of genomic segments, we used the "local *z*-score" function as implemented in *regioneR*. Z-scores are calculated as the distance between the permuted (or expected) and the observed overlap between a given pair of interval sets (see Figure A in S1 File). In this process one set of intervals is fixed (*e*.*g*. TADs boundaries) and the other (*e*.*g*. LD-blocks boundaries) is shifted using a selected window size (*e*.*g*., 500 kb) sliding over the fixed intervals set; at each shift, the overlap is evaluated and scaled by standard deviations to obtain a z-score value. Following this procedure, we performed a permutation test and computed *p*-values and *z*-scores iteratively with shifted positions, 500 kb in 5' and 3' directions with respect to a focal position. Plotting average *z*-scores versus shifted positions, one can visualize how the value of the *z*-score changes when moving away from the focal site: A peak at the centre of the fixed genomic intervals indicates that the association is dependent on matched boundaries, while a flat profile indicates diffuse association.

## Results

### Association of recombination rate with TADs and isochores

**Fig 1:**
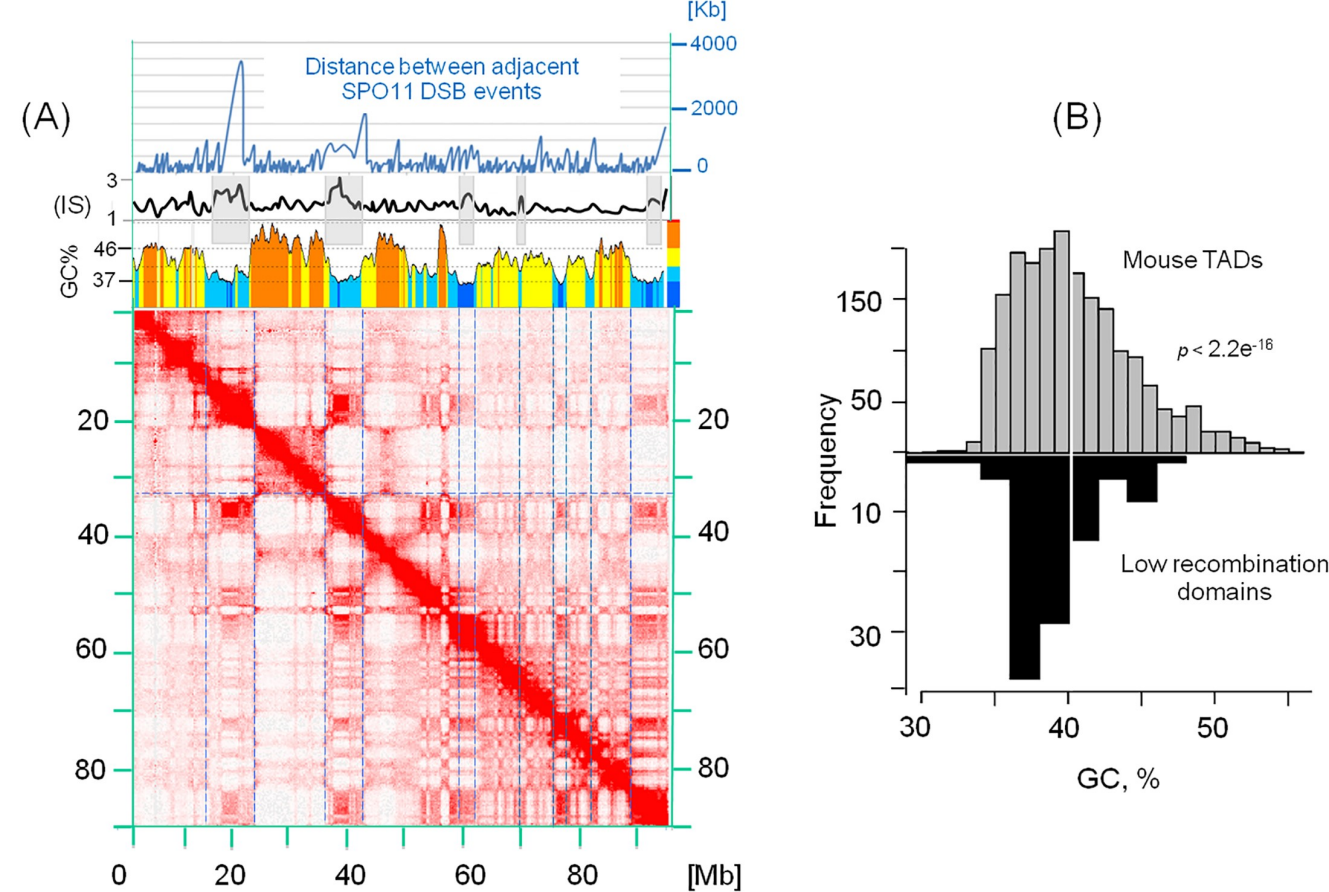
Spatial distribution of low recombination domains, TADs/LADs and isochores. (A) The heat-map of chromatin interactions in mouse chromosome 17 from round spermatids is aligned to the profile of cold spot information score (IS) (top blue curve with grey banded cold spots) and to the corresponding compositional profile (the multi-coloured profile). The GC profile is drawn from mouse mm10 genome assembly with a sliding window of 300 Kb (with steps of 30 Kb). Increasing GC isochores are represented by deep blue (L1), light blue (L2), yellow (H1) and orange (H2) (see colour code bar left to the GC profile). The top panel represents the distance between adjacent SPO11 DSB events; large distances are typical of LRDs. Blue dashed lines across the heat-map delimit LRDs. (B) GC% of ES cells TADs (top) and LRDs (bottom).

To test for the association between TADs and recombination domains in mice, we used the Hi-C map constructed from round spermatids and the map of low recombination domains (LRDs) constructed from the DO mice cohort. Fig 1A shows a remarkable overlap of LRDs, low GC-isochores and chromatin domains from mouse chromosome 17. GC-poor chromosomal domains, which are frequently attached to the interphase lamina (LADs) [38,40,41], coincide with regions of reduced recombination. This is further confirmed by the larger distance between consecutive DSBs in GC-poor TADs (Fig 1A); similar results are obtained with embryonic stem cells (Figure B in S1 File). This observation is not limited to the mouse chromosome 17, a histogram of GC% of LRDs from all chromosomes (Fig 1B) shows that they are strongly biased towards low GC values compared to overall GC% distribution of TADs (mean difference *t*-test; *p*-value < 2.2e^-16^). This indicates that the GC-poor TADs *vs*. LRDs correspondence is a genome wide feature.

**Fig 2:**
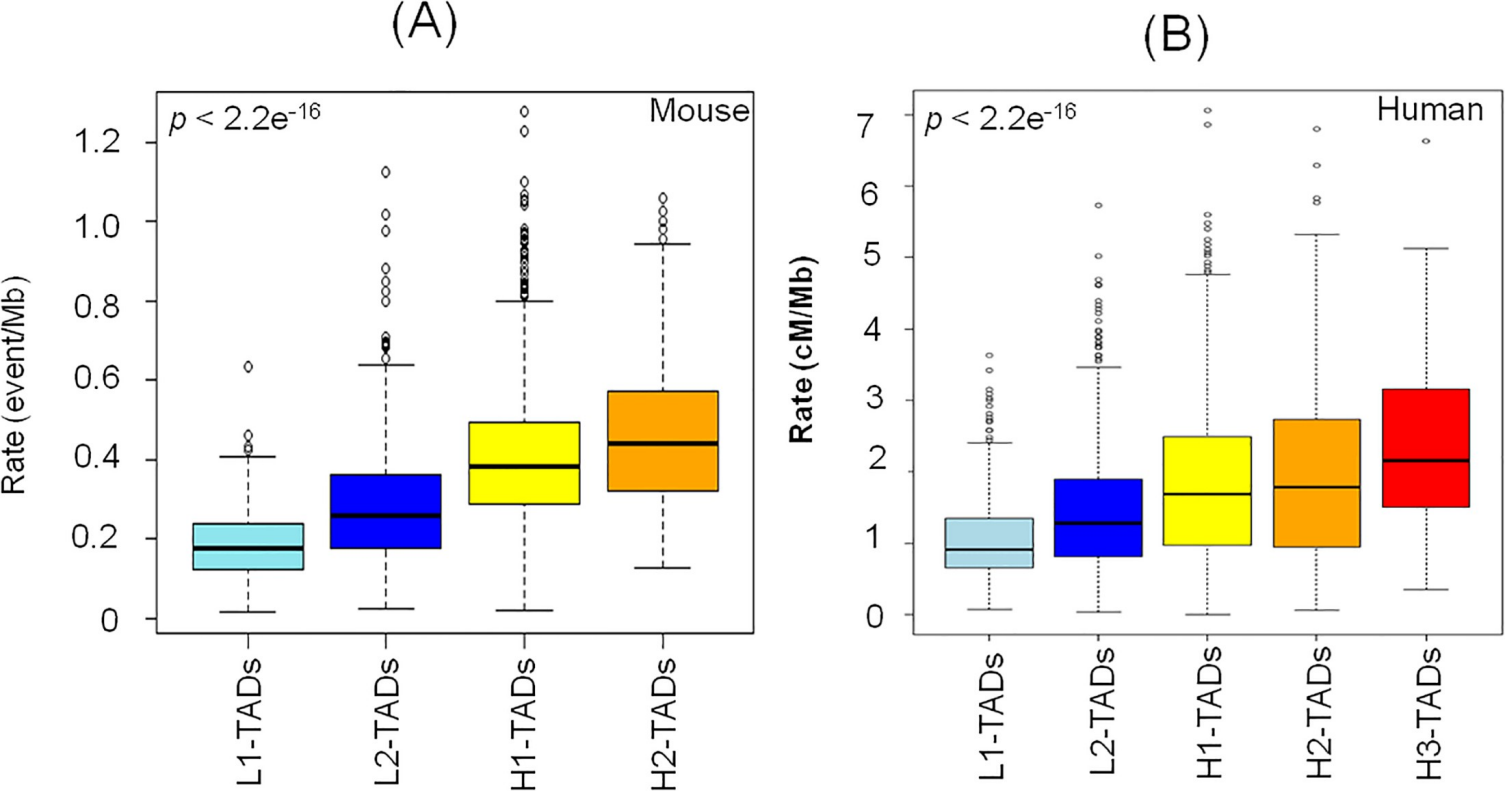
Correlation between recombination rate and isochore or GC level of TADs. Box plots showing the increase in recombination rate with TAD GC level in mouse (A) and human (B). TADs are grouped in GC classes according to isochore boundaries: L1 (33–37%GC), L2 (37–41%GC), H1 (41–46%GC), H2 (46–53%GC) and H3 (53–59%GC). H3 Isochores are known to be very scarce or absent in mouse [46, 58].

Corroborating the results of Fig 1A and 1B, we also observe a significant positive correlation (*p*-value < 2.2 e^-16^, Pearson correlation test) between recombination rate and TAD GC% in mouse (Fig 2A) and human (Fig 2B).

### Linkage disequilibrium blocks match TADs and LADs

**Fig 3:**
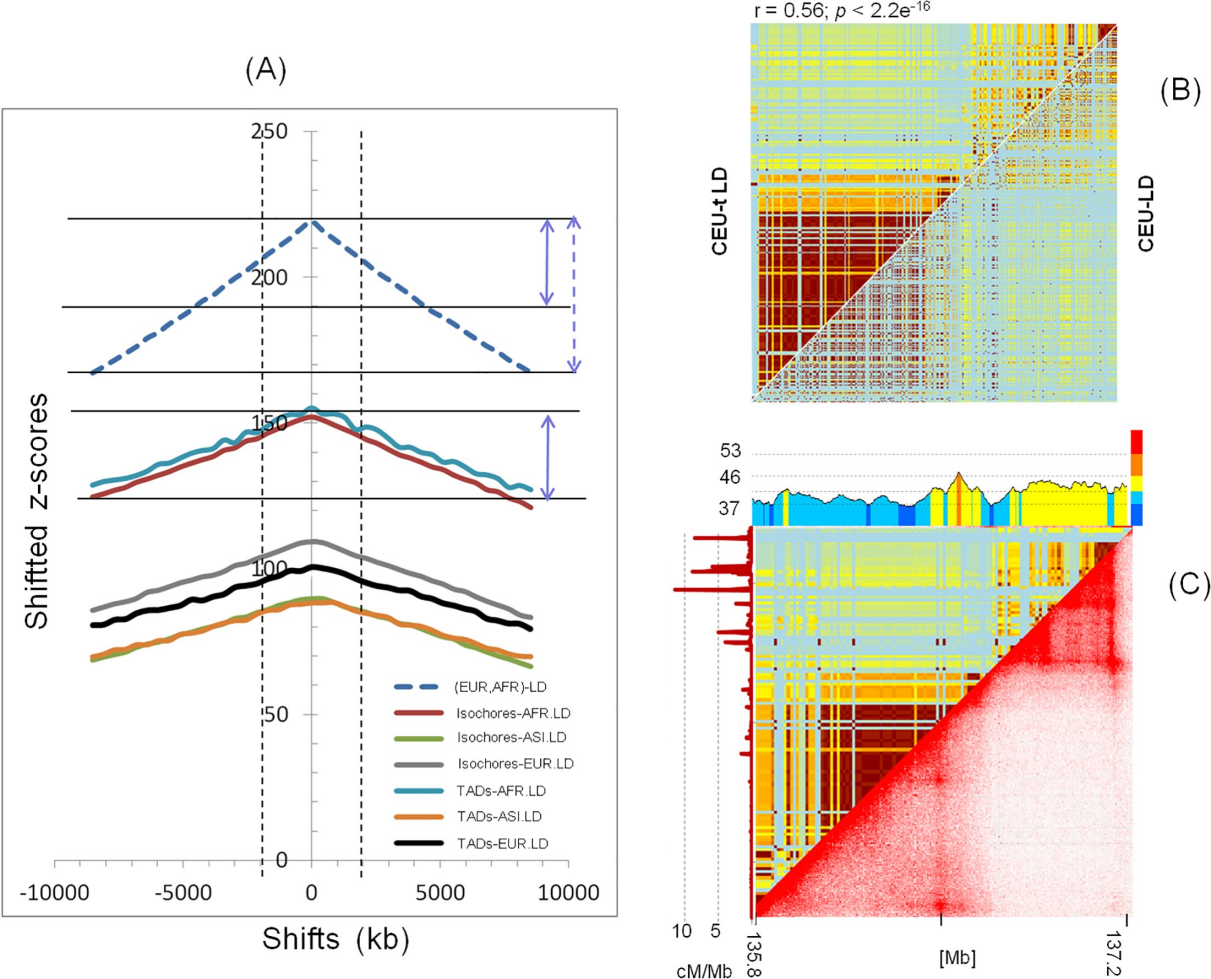
Concordance between boundaries of TADs, Isochores and LD-blocks. (**A**) Shifted *z*-score (on the y-axis) changes when moving away from the focal point (x-axis): the peak at the centre indicates that the association is dependent on the genomic coordinates, while a flat profile indicates that the association is either absent or boundary independent. Abbreviations: ASI-LD, AFR-LD and EUR-LD correspond to Asian, African and European linkage disequilibrium blocks, respectively. Black and blue arrows point to the difference in *z*-scores drops between EUR-LD *vs*. AFR-LD and isochores or TADs *vs*. AFR-LD, indicating a weaker drop of *z*-scores in the latter compared to the former. The arrows indicate ~50% drop in *z*-score compared to the reference (EUR-LD, AFR-LD). (**B**) A case study of matched LD and TADs boundaries. The heat-maps of conventional (bottom right triangle) and topological LD (top left triangle) is generated for a region of chromosome 2 (chr2:135.8–137.2 Mb) from the CEU subsamples of the human 1k genomes project (right). (**C**) The aligned heat maps of isochores, TADs and tLD-blocks from the same region. Left vertical profile (brown line) represents recombination frequency in cM/Mb.

The results of Fig 1A suggest that the co-localisation of TADs and LRDs boundaries may be a general feature of mammalian genomes. Because regions of low recombination tend to have elevated levels of linkage disequilibrium, we expected that LD-blocks should also coincide with TADs. To test this hypothesis in human, we evaluated the statistical significance of co-localisation of LD-blocks, TADs, and isochores. The genome wide statistical tests for overlap between TADs, isochores and LD-blocks were all found to be highly significant (Figure C in S1 File); all *p*-values < 0.001. To assess the strength of boundary sharing between LD-blocks and TADs, we first established a reference by calculating the *z*-score profile of shared LD-blocks between European and African populations. The *z*-score profiles for shared TADs and LD-blocks boundaries are plotted with respect to this reference (Fig 3A).

The *z*-score profiles (Fig 3A) clearly indicate a relative concordance between TADs, isochores and LD-blocks boundaries. Taking into account the reference *z*-score profile of LD-blocks match between African and European, the match between TADs/isochores and LD-blocks is almost half as strong compared to the adopted reference. Interestingly, the peak of the *z*-score profile is most pronounced for the ancestral African population (see Discussion).

The regional co-variance of TADs, isochores and recombination domains or LD-blocks can be visualized using a LD heat-map based on a measure called topological LD (tLD); tLD is based on genealogical rather than allelic clustering (see [59] for more details). As expected, the two LD heat maps (Fig 3B) are very similar, pairs of tLD and LD values are significantly correlated (r = 0.56; p-value < 2.2 e-^16^), with the advantage of a better contrasted signal in tLD graphical visualisation. As shown in Fig 3C, TADs and LD-blocks boundaries appear to match each other and the two match the isochore boundaries, probably constrained by flanking LADs (Fig 3C and Figure D in S1 File) or by specific association to meiotic synaptonemal axes in leptotene cells. Recombination frequency is also compartmentalized, the GC-rich TAD (Fig 3C) has clearly higher recombination frequency, hence fitting the expectation from the positive correlations between GC-content and recombination reported in Figs 1 and 2. We notice that the loop boundary is invariably located in the GC-rich area covering LCT and MCM6 genes in the seven cell types analyzed (Figure D in S1 File). This may be expected because TADs tend to be conserved among tissues, for instance, 65% to 72% of TAD boundaries are common between human embryonic stem cells and IMR90 fibroblasts [37]. For more examples of LD vs. TAD concordance see Figure E in S1 File.

### Recombination machinery components and TADs

An independent way of ascertaining the above observations (Figs 1 and 2) consists in using a set of genomic hallmarks of recombination. In the case of mouse, we analyzed the genomic binding distribution of key recombination proteins mapped around DSB sites [54,55]. These were found to be positively correlated with recombination rate and with the GC content of TADs (Table 1 and scatter plots in Figure A in S1 File). Weak to moderate positive correlations are observed, all statistically significant (*p*-values < 2.2 e-^16^). The strength of the correlation coefficients and their significance do not change if different PRDM9 alleles or different mice strains are considered (data not shown).

**Table 1:** Pearson correlation coefficients between genomic features. “RF” refers to recombination frequency (recombination events per Mb) in B6 mouse strains. Correlation coefficients are all significant at *p*-values less than 2.2 e-^16^.

	SPO11	PRDM9	H3K4me3	DMC1
**TADs GC%**	0.38	0.32	0.35	0.20
**RF**	0.38	0.34	0.22	0.33

## Discussion

### Interphase-leptotene chromatin reorganization

In mice and humans, spermatocytes begin to enter leptotene, the first stage of meiotic prophase, and later in this stage chromosomes are re-organized in alternating domains of higher and lower DSB activity [60,61]. This heterogeneity is not yet well documented, although an inverse relation between loop size and axis length was reported [62,63]. This configuration is apparently quite different from the interphase chromatin, where loops are heterogeneous in size and nucleotide composition, and about 40% of the genome is associated with the nuclear envelope [38,41], instead of anchoring along the synaptonemal axis as in leptotene. These differences are contrasted by some similarities, in fact, independently of cell type or lineage, cohesin and condensin have an architectural role in the organization of interphase chromosomes and similar roles have been proposed for cohesin and the related condensin complexes in meiotic and mitotic chromosomes [64–65]. This begs an interesting question: can the pre-existing higher order chromatin structure in the interphase of progenitor germ cells or pre-leptotene spermatocytes be related to that of early leptotene? It was previously anticipated that meiotic prophase chromatin features may have evolved directly from the later stages of the mitotic program [26], a hypothesis reminiscent of the recently proposed “topological memory” [66,67]. The spatial correlation between the recombination rate profile and the loop structure of spermatids and ES cells argues in favour of this link (Fig 1 and Figure B in S1 File). Moreover, shorter loops are biased towards GC-rich genomic domains (see Fig 1A and [41]), as seen in interphase chromatin and in consistence with the smaller size of the loops attached to the synaptonemal complex in the telomeric and subtelomeric regions of mouse [68], known to be GC- and gene-rich [69–71]. The size heterogeneity of meiotic loops can be understood from the lower density of DSBs in LADs (Fig 1A). This is also evident from the distance between adjacent DSBs, which is larger in GC-poor than in GC-rich TADs (Fig 1A top panel), reflecting non-homogenous attachment of loop bases to the components of the synaptonemal axis.

These similarities suggest that chromatin loops of leptotene chromosomes are, at least in part, inherited from the interphase of pre-leptotene spermatocytes. This transfer can be mediated by DNA compositional constraints, synaptonemal complex, active (*e*.*g*. H3K4me3, H3K9ac and H3K4me2) and repressive (*e*.*g*. H3K9me3, H3K27me3, H3K9me2) chromatin marks ([72] for a review). Incidentally, Lamin B1, a component of LADs, is also associated with PRDM9-binding depleted regions [73]. These combined factors could maintain genomic regions in a “poised” folding state participating later in the assembly of chromosome axes and loops.

Summarizing, DSBs 1) are influenced by chromatin modifiers and DNA binding of transcription factors influences DSB density [74,75]; 2) they occur in regions of accessible chromatin (GC-rich) that are present in mitotic as well as in meiotic cells [76]; 3) they typically take place in loop sequences [77] and their chromosomal distribution can be affected by the sequence composition of loops, as shown in Fig 1. Good correlations between recombination machinery components and GC% of TADs (Table 1 and Figure F in S1 File) confirm the observations mentioned above and suggest the existence of compositional constraints on the formation of chromatin loops and DSB-suppressed domains.

### Mammalian recombination and chromatin landscape

**Fig 4:**
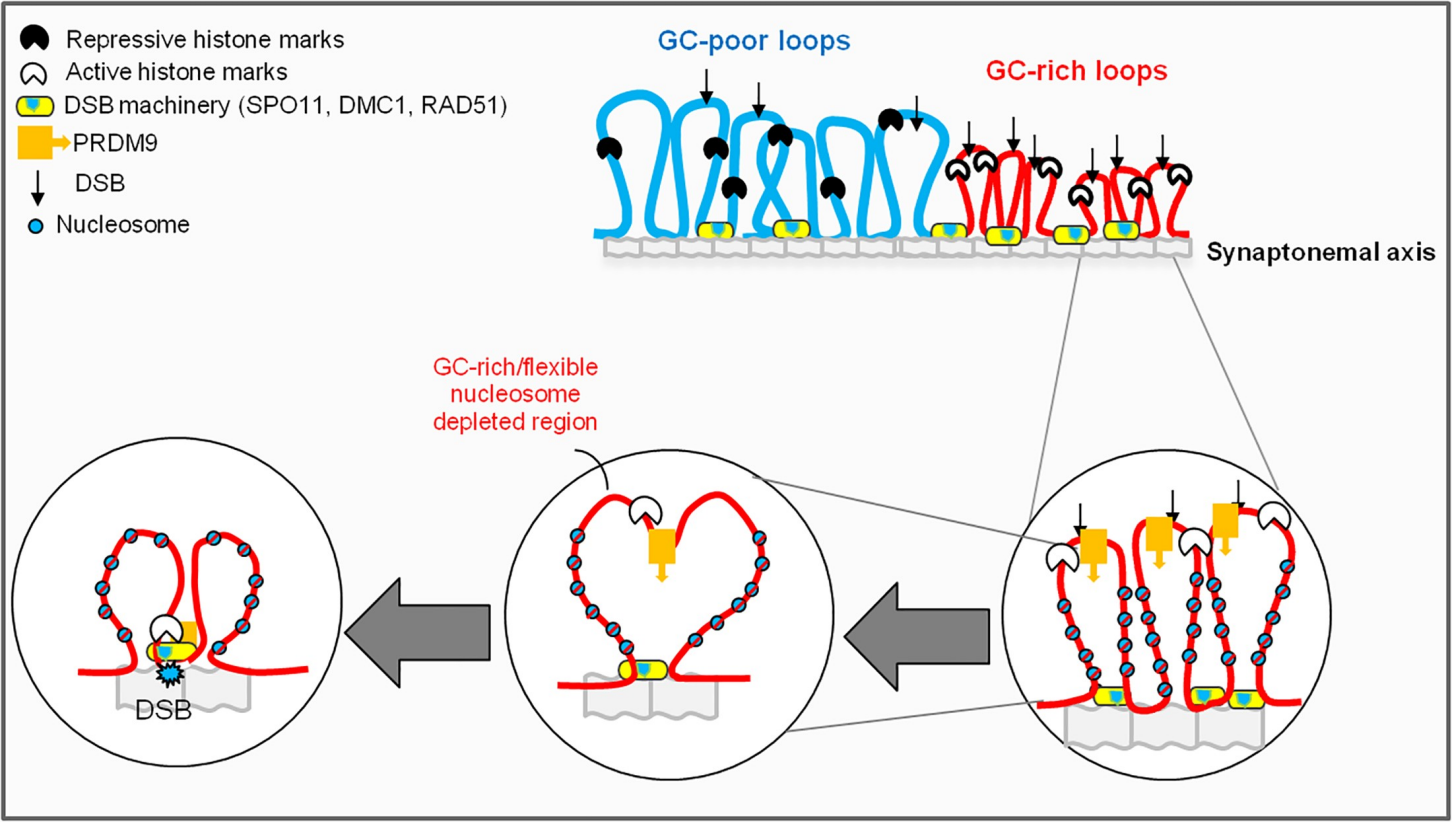
Cartoon depicting a model of chromatin fibre state in late leptotene-early zygotene cells. Only one homolog and one sister chromatid are shown. Not all structural components of the chromosome axis are represented. In the present model, GC-rich gene dense regions are endowed with shorter loops compared to AT-rich regions. Loop formation is driven by local flexibility of the DNA fibre, histone modification and nucleosome density. GC-poor chromosomal regions are enriched for H3K9me2 (histone H3 lysine9 dimethyl), H3K9me3, Lamin B1 (not drawn), and depleted for PRDM9-binding. In species endowed with PRDM9, this protein binds to specific GC-rich DNA motifs and promotes histone methylation (H3K4me3 and H3K36me3). Recruitment of proteins required for the formation of DSBs by SPO11 occurs in the presence of the DSB repair proteins DMC1 and RAD51 located at the loop base in the vicinity of the synaptonemal axis. PRDM9 may promote the recruitment of SPO11 to the loop base, but in the case of PDRM9-less genomes (like Canidae), SPO11 needs to be recruited by other bridging mediators, in concert with local sequence dependent DNA shape and affinity. The recruitment of relatively GC-rich and nucleosome depleted segments of the loop to the synaptonemal axis is facilitated by their higher bendability. The relative rigidity of the AT-rich sequence in GC-poor domains may preclude the formation of shorter loops due to reduced flexibility.

At shorter scales, nucleosome formation generally restricts the accessibility of proteins to DNA, including SPO11. Meiotic DSBs are thought to be introduced on chromatin loop regions that transiently interact with the lateral elements of the synaptonemal complex, where SPO11 is attached [61,77,78] (see also Fig 4). This suggests that these chromatin domains may contain nucleosome-depleted regions (GC-rich open chromatin) [55,78–79] (see Fig 4). Thus, the local recombination effects are expected to be stronger in GC-rich TADs, where loops are shorter, nucleosomes are spaced, PRDM9 (when present) and CTCF/CTCFL binding sites are enriched [54].

DSB formation involves three major steps (reviewed in [80,81]): I), SPO11 is loaded to DNA either at accessible chromatin regions and/or through recruitment via other partners; II) DSB sites are tethered to the synaptonemal axis; III) SPO11 is inducing DSB formation. At each of these steps, local flexibility, stiffness and binding affinity of the DNA fibre can influence DSB occurrence. The second step can impose an additional tension on the DNA loop (Fig 4); such tension is expected to be less effective in short (close to the synaptonemal axis) and GC-rich (more flexible) loops. If regulation of chromatin loop size comprises a major mechanism for global regulation of overall crossover frequency [82], then the basic compositional design of loop DNA sequences should interfere with such mechanism. A model representing these features and their relation to compositional constraints that can shape DNA bendability and flexibility [83] is recapitulated in Fig 4.

Two aspects related to the model presented in Fig 4 should be highlighted: (1) as in mitotic chromosomes, meiotic loops are stabilized by the dynamic binding of cohesin and meiotic insulators (*e*.*g*. CTCF) [84,85]. Of note, recent studies showed that BORIS (or CTCF-like), a genomic neighbour of SPO11 (CTCF-like and SPO11 are located at the base of same interphase chromatin loop [86]), is present in male germ cells during and after meiosis, and may interact with at least one of the meiosis-specific subunits of cohesin complexes, hence contributing to a progressive re-establishment of genome architecture in haploid post-meiotic round spermatids [87,50]; (2) although a functional PRDM9 homolog is reported to be missing in dogs [32], LRDs distribution across the dog genome recapitulates the major features of the mouse recombination map, in particular, recombination appears to be directed to gene promoters and CpG islands [34]. This is in agreement with the observation that isochores and TADs are conserved between human, dog and mouse [36,39,41,88].

Together, our results indicate that the recombination machinery may follow the principles of 3D DNA fibre organization in their selection of recombination partners, a view in line with damage-induced DSBs experiments where DSBs are believed to be primarily dictated by genome shape and nuclear position [89,90]. Indeed, chromatin in GC-rich regions was early reported to be more expanded relative to chromatin in AT-rich regions [91] and, chromosome axes are straight in R bands and coiled in G bands [92].

### LD-blocks and chromatin neighbourhoods

The concordance between TADs, isochores and LD-blocks revealed in this work has two consequences: (1) it suggests that regional variation of recombination is topologically defined thanks to an underlying compositional and epigenetic framework (common genomic code). GC-rich domains are recombinogenic, which may explain the smaller size or, in other worlds, the fragmentation of GC-rich LD-blocks (Figure G in S1 File). As a result, shared LD-blocks between Europeans, East Asians and Africans, are significantly larger in size (Figure H in S1 File); their lower recombination rate is congruent with the observation that strong LD-blocks tend to be GC-poor [8]; (2) the strong LD between a pair of SNPs in GC-rich recombinogenic TADs may hint to functional chromatin contacts maintained by purifying selection. A reduced recombination rate between target genes and their regulatory elements may come with a selective advantage when linkage between genes and regulatory elements needs to be maintained as a single inheritance or evolutionary unit. The ability of LD-blocks to encompass long range SNP interactions in regions with enhanced intra-loop contacts highlights how analyses of allelic chromatin topology can help to infer mechanisms by which SNPs associate with disease and traits [93]. Comparative haplotype mapping of chromatin interactions is one possible approach; another possibility is to assess disease association of disrupted CTCF-mediated interactions by examining linkage disequilibrium between the SNPs residing in CTCF motifs and genome wide disease-associated SNPs [94].

As far as the evolutionary age of LD-blocks is concerned, chromosomal segments have been broken down by repeated meioses along human evolution. Assuming that the overall level of LD is closer to recombination-drift equilibrium in the ancestral African sub-population than in the derived European and Asian sub-populations, as also expected from homozygosity runs (ROH) being longer in Europeans and Asians compared to Africans [95]. Our observation of a distinct *z*-score profile in Africans on one hand and in Europeans and Asians on the other (Fig 3A), is consistent with this assumption. LD-blocks of much older origin are generally long and GC-poor, they may persist in the population due to regionally low recombination rates. Overall, these domains are enriched for structural variations, in particular deletions [96,34] that can disrupt local chromosome synapsis, which will in turn suppress recombination between miss-aligned chromosomal loci [34].

## Conclusion

Although the occurrence of hot spots of recombination and its relation to open chromatin was suspected many decades ago, the recent observation of the correspondence between isochores and TADs, and the large number of available genomic maps allowed us to re-visit the recombination landscape in mammals and connect it to chromatin architecture. We have shown that the majority of low recombination domains coincides with pre-leptotene constitutive LADs (the GC-poorest, transcriptional repressed isochores). Our data suggests that the trade-off between major determinants of the genomic code, namely DNA sequence constraints and the associated modified or native protein binding partners, can affect the conformation of the chromatin fibre, primarily determined by its own stiffness and flexibility. LRDs and LD-blocks are shown to significantly, albeit weakly, match TADs and isochores. Moreover, loading and binding affinities of key determinants of meiotic recombination hot spots (PRMD9, SPO11, DMC1, H3K4me3) are positively correlated with TADs GC%. Combined, these observations suggest that DSB and recombination frequencies are associated with similar compositional and epigenetic features that constrain the distribution of chromatin in the interphase nucleus. Pre-meiotic TADs and sub-TADs and their sequence design (*e*.*g*. oligonucleotide frequencies) might represent a structural template, within which meiotic loops organize themselves, evoking a component not yet taken into consideration in the “topological memory” paradigm.

## Supporting information

S1 File Supplementary figures. Figure A. Schematic representation of “regioneR” approach. We first perform permutation test by creating 1000 randomizations of set-2 to test if the overlap with set-1 is more than expected, the output is then stored in the object “pt”. We can then plot the “pt” object; plot(pt) will create a plot with the distribution of the permuted regions set and the original one (set-1). In grey the number of overlaps of the randomized regions, clustering around the black bar that represents the mean and in green the number of overlaps of the original region set-1, which is much larger than expected. The red line denotes the significance limit. The second step is not dependent on the number of permutations since it will use the results from the previous step, the permutation test result object “pt”. For each individual shift (s_i_) of segments from set-2, z-score, a measure of the strength of the association/overlaps with intervals from set-1, is calculated as the distance between the expected (the mean of permuted) and the observed association values, scaled by standard deviation over the sample (set-1). We can test if the association between the two region sets is dependent on their boundaries by using the “localZScore” function. Shifted z-scores peak at the centre of intervals from set-1 indicates that boundaries from this set and those from set-2 tend to match each other; while a flat profile indicates diffuse association. The pictured z-scores shape is indicative of deviation from the null hypothesis (boundaries mismatch). “R” command syntax from “regioneR” package is indicated in red rectangles. Figure B. Spatial match between Low Recombination Domains (LRDs), TADs and Isochores. The heat-map of chromatin interactions in mouse embryonic stem cells of chromosome 17 is from Bonev *et al*. 2017. Other annotations are as in Fig 1. Fig C. Association analysis of genomic regions (TADs and LD-blocks) based on permutation test. In grey the number of overlaps of the randomized regions of TADs, they all cluster around the black vertical lines representing means; green vertical lines represent the number of overlaps of randomized TADs with the original region of LDs sets, these values are much larger than expected (see horizontal double arrow lines). The red vertical line denotes the significance limit. Human LDs from African (ARF), European (EUR) an East Asian (ASI) overlap significantly with TADs from human ESC cells and isochores. Figure D. Chromatin loop boundary stability along a case study locus (chr2:135.8–137.2 Mb). Similar topology is noticeable between the different cell types, the green band indicates the conserved boundary which overlaps with LCT (Lactase) and MCM6 (Minichromosome maintenance complex component 6) genes; the introns of the latter contain enhancers of the former, indicating a functional and possibly a topological coupling. Figure E. Chromatin loops boundaries annotated in IMR90 fibroblasts from [39]. The black bars indicate TADs boundaries. Under the bars, LD-blocks and TADs genomic coordinates are given. In all cases shown here the overlap between LD (from African population) and loops is more that 70%. Fig F. Scatter plot representing correlations between recombination frequency and GC% of TADs (on the x-axis) on one hand and the PRMD9, SPO11, DMC1 and H3K4me3, occupancy frequencies on the other (on the y-axis). Figure G. Scatter plot representing correlations between LD-blocks GC%, LD-block size and recombination rate. “LD-block R” is the recombination rate per LD-block. Figure H. Box plot showing significant (p-value < 2. 2e-16) increase of shared LD-blocks size; median = 1.3 Mp for “Shared by all” LD-blocks *vs*. 0.9 Mb for “Not in (EUR+AFR)”.
(PDF)
