# Supplementary material for: A common genomic code for chromatin architecture and recombination landscape
Source: PLoS One. 2019 Mar 13;14(3):e0213278. doi: 10.1371/journal.pone.0213278 (PMC6415826; doi:10.1371/journal.pone.0213278)
Supplement: S1 File — Figure A. Schematic representation of “regioneR” approach. We first perform permutation test by creating 1000 randomizations of set-2 to test if the overlap with set-1 is more than expected, the output is then stored in the object “pt”. We can then plot the “pt” object; plot(pt) will create a plot with the distribution of the permuted regions set and the original one (set-1). In grey the number of overlaps of the randomized regions, clustering around the black bar that represents the mean and in green the number of overlaps of the original region set-1, which is much larger than expected. The red line denotes the significance limit. The second step is not dependent on the number of permutations since it will use the results from the previous step, the permutation test result object “pt”. For each individual shift (si) of segments from set-2, z-score, a measure of the strength of the association/overlaps with intervals from set-1, is calculated as the distance between the expected (the mean of permuted) and the observed association values, scaled by standard deviation over the sample (set-1). We can test if the association between the two region sets is dependent on their boundaries by using the “localZScore” function. Shifted z-scores peak at the centre of intervals from set-1 indicates that boundaries from this set and those from set-2 tend to match each other; while a flat profile indicates diffuse association. The pictured z-scores shape is indicative of deviation from the null hypothesis (boundaries mismatch). “R” command syntax from “regioneR” package is indicated in red rectangles. Figure B. Spatial match between Low Recombination Domains (LRDs), TADs and Isochores. The heat-map of chromatin interactions in mouse embryonic stem cells of chromosome 17 is from Bonev et al. 2017. Other annotations are as in Fig 1. Fig C. Association analysis of genomic regions (TADs and LD-blocks) based on permutation test. In grey the number of overlaps of the randomized regions [file pone.0213278.s001.pdf]

Figure A

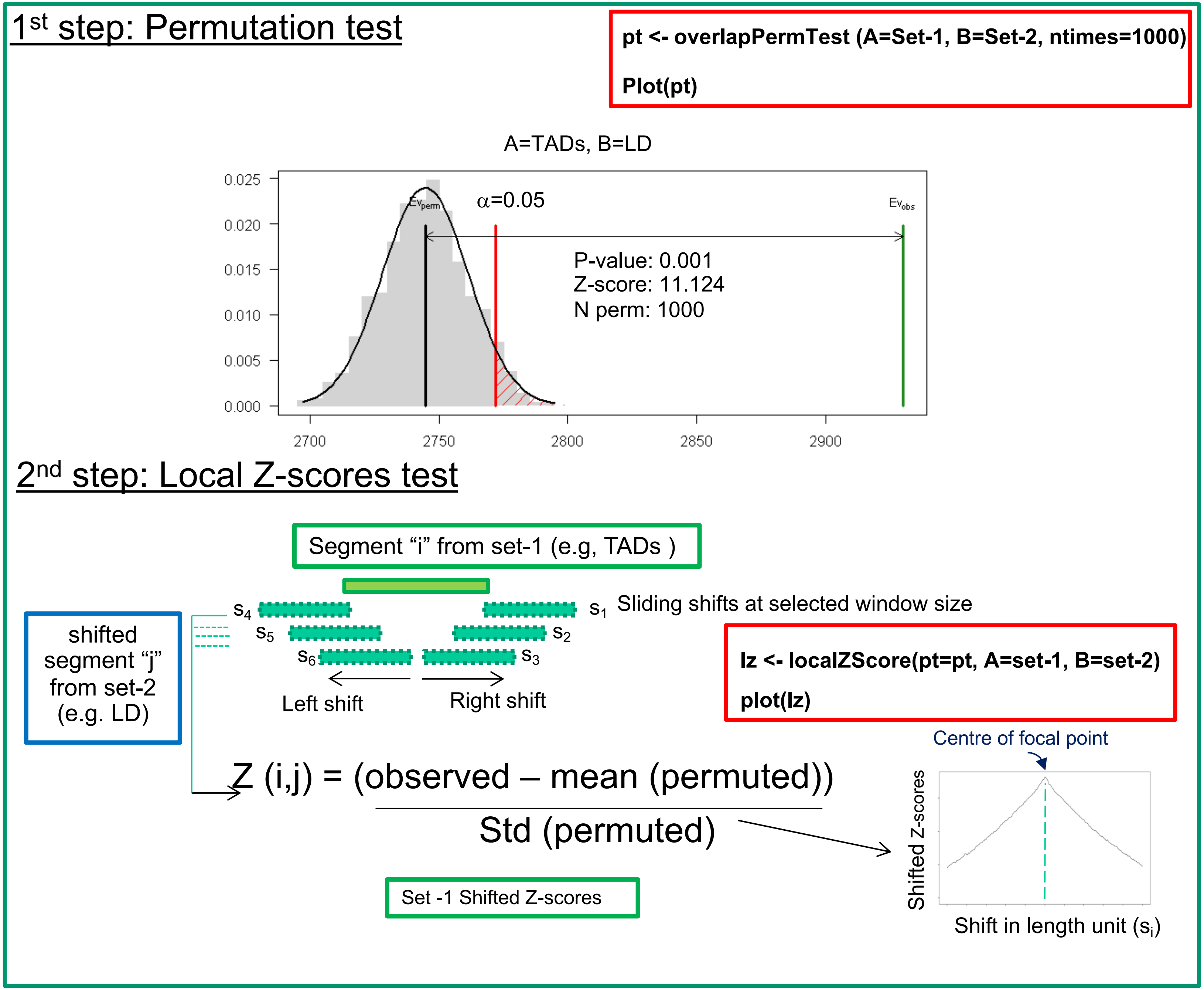

1st step: Permutation test
pt <- overlapPermTest (A=Set-1, B=Set-2, ntimes=1000)
Plot(pt)
A=TADs, B=LD
α=0.05
Ev_perm
Ev_obs
P-value: 0.001
Z-score: 11.124
N perm: 1000
0.025
0.020
0.015
0.010
0.005
0.000
2700
2750
2800
2850
2900
2nd step: Local Z-scores test
Segment "i" from set-1 (e.g, TADs)
shifted segment "j" from set-2 (e.g. LD)
s4
s5
s6
s1
s2
s3
Sliding shifts at selected window size
Left shift
Right shift
lz <- localZScore(pt=pt, A=set-1, B=set-2)
plot(lz)
Z (i,j) = (observed – mean (permuted)) / Std (permuted)
Centre of focal point
Shifted Z-scores
Shift in length unit (si)
Set -1 Shifted Z-scores

Figure B

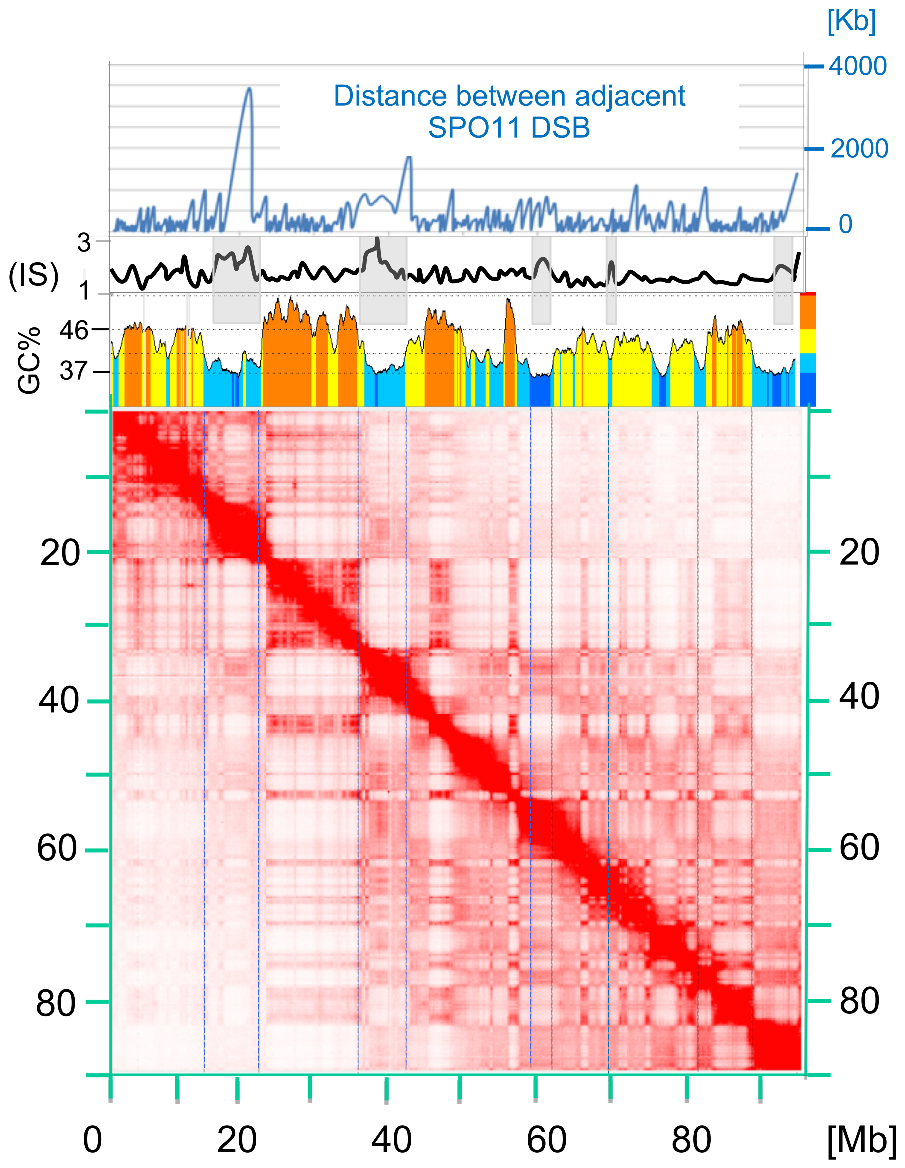

[Kb]
4000
2000
0
Distance between adjacent
SPO11 DSB
3
(IS)
1
GC%
46
37
20
40
60
80
0
[Mb]

Figure C

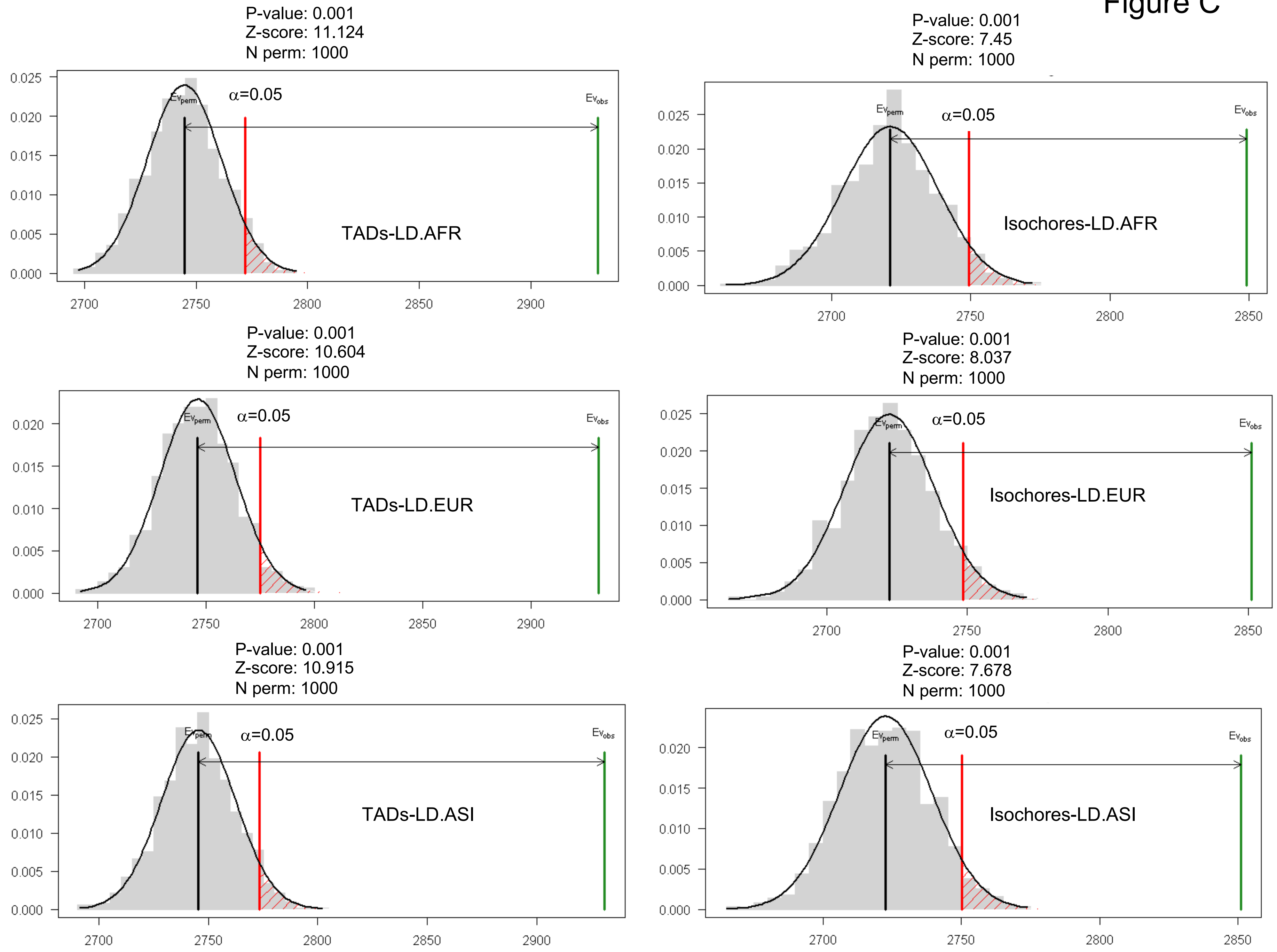
P-value: 0.001
Z-score: 11.124
N perm: 1000
α=0.05
Ev_perm
Ev_obs
TADs-LD.AFR
P-value: 0.001
Z-score: 7.45
N perm: 1000
α=0.05
Ev_perm
Ev_obs
Isochores-LD.AFR
P-value: 0.001
Z-score: 10.604
N perm: 1000
α=0.05
Ev_perm
Ev_obs
TADs-LD.EUR
P-value: 0.001
Z-score: 8.037
N perm: 1000
α=0.05
Ev_perm
Ev_obs
Isochores-LD.EUR
P-value: 0.001
Z-score: 10.915
N perm: 1000
α=0.05
Ev_perm
Ev_obs
TADs-LD.ASI
P-value: 0.001
Z-score: 7.678
N perm: 1000
α=0.05
Ev_perm
Ev_obs
Isochores-LD.ASI
0.000
0.005
0.010
0.015
0.020
0.025
2700
2750
2800
2850
2900

Figure D

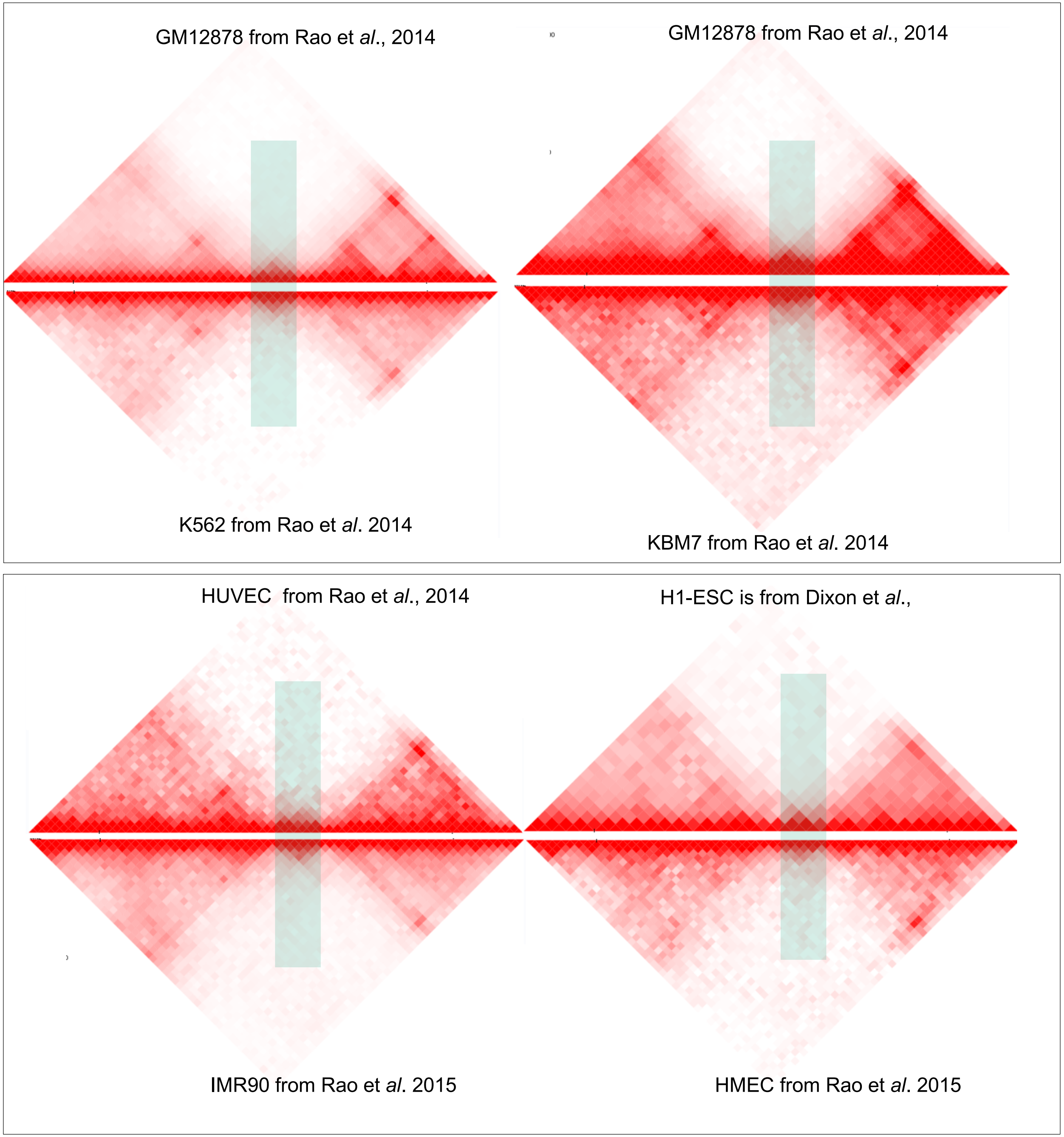

GM12878 from Rao et *al*., 2014
GM12878 from Rao et *al*., 2014
K562 from Rao et *al*. 2014
KBM7 from Rao et *al*. 2014
HUVEC from Rao et *al*., 2014
H1-ESC is from Dixon et *al*.,
IMR90 from Rao et *al*. 2015
HMEC from Rao et *al*. 2015

Figure E

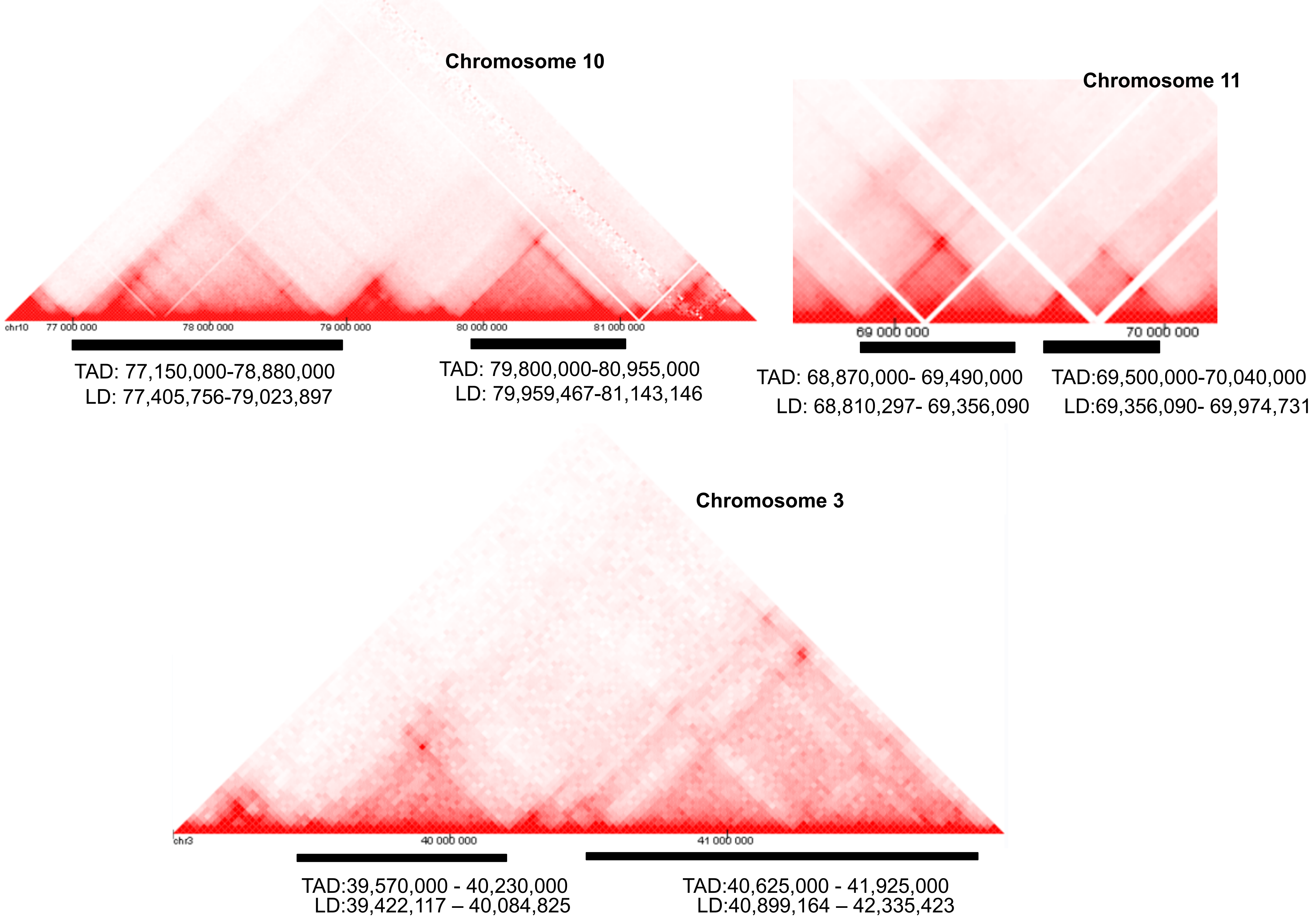
Chromosome 10
chr10
77 000 000
78 000 000
79 000 000
80 000 000
81 000 000
TAD: 77,150,000-78,880,000
LD: 77,405,756-79,023,897
TAD: 79,800,000-80,955,000
LD: 79,959,467-81,143,146
Chromosome 11
69 000 000
70 000 000
TAD: 68,870,000- 69,490,000
LD: 68,810,297- 69,356,090
TAD:69,500,000-70,040,000
LD:69,356,090- 69,974,731
Chromosome 3
chr3
40 000 000
41 000 000
TAD:39,570,000 - 40,230,000
LD:39,422,117 – 40,084,825
TAD:40,625,000 - 41,925,000
LD:40,899,164 – 42,335,423

Figure E (continued)

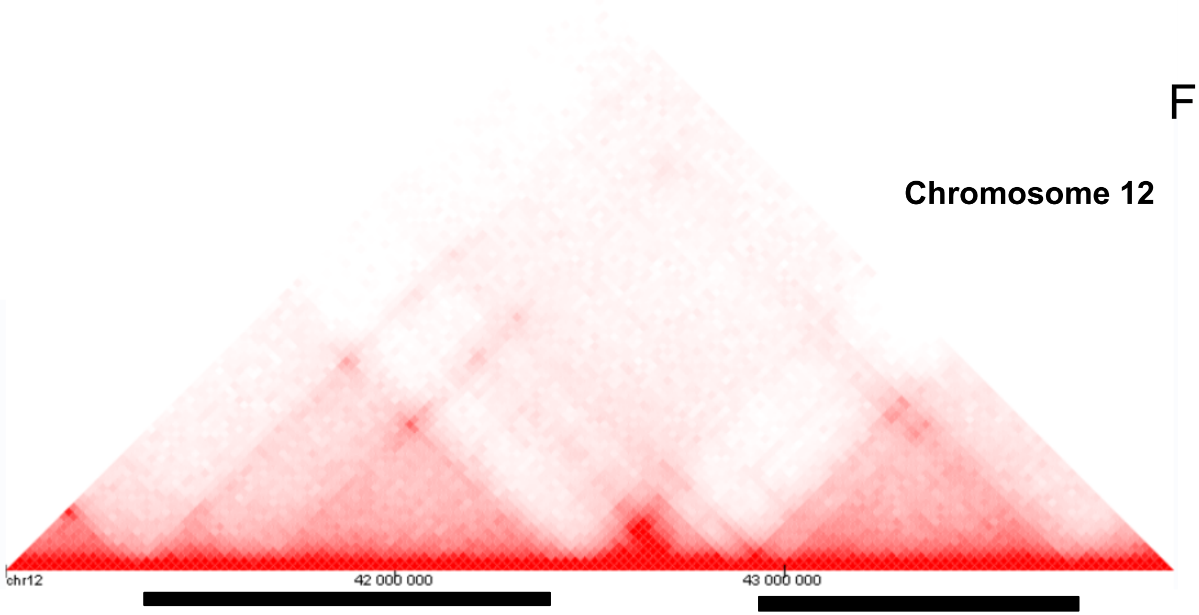

TAD: 41,330,000-42,420,000
LD: 41,198,874- 42,347,363

TAD:42,870,000-43,740,000
LD: 43,074,644-43,983,111

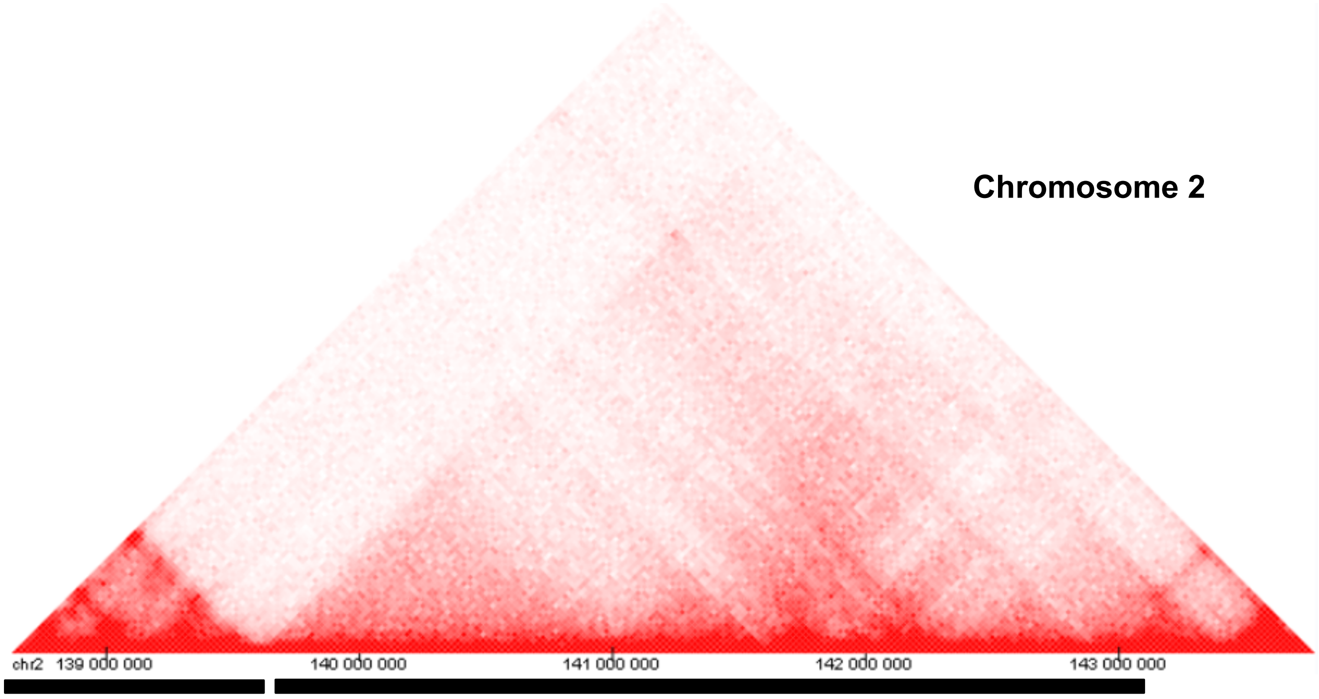

TAD: 138,650,000 - 139590000
LD: 138,880,563 -139,811,930

TAD: 142,920,000 – 143,750,000
LD: 142,793,102 – 143,926,523

Figure F

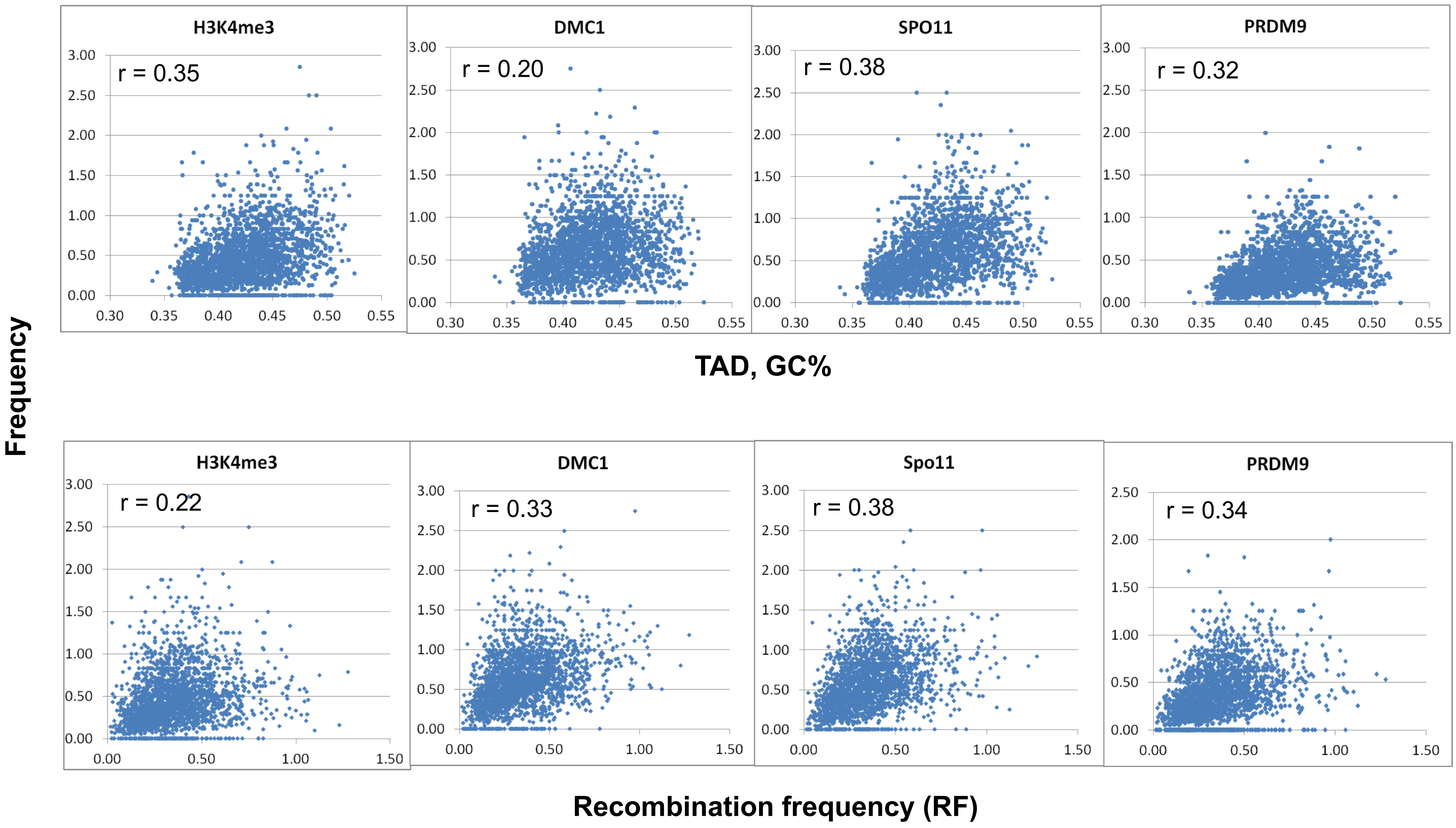
Frequency
H3K4me3
r = 0.35
DMC1
r = 0.20
SPO11
r = 0.38
PRDM9
r = 0.32
3.00
2.50
2.00
1.50
1.00
0.50
0.00
0.30
0.35
0.40
0.45
0.50
0.55
TAD, GC%
H3K4me3
r = 0.22
DMC1
r = 0.33
Spo11
r = 0.38
PRDM9
r = 0.34
0.00
0.50
1.00
1.50
Recombination frequency (RF)

Figure G

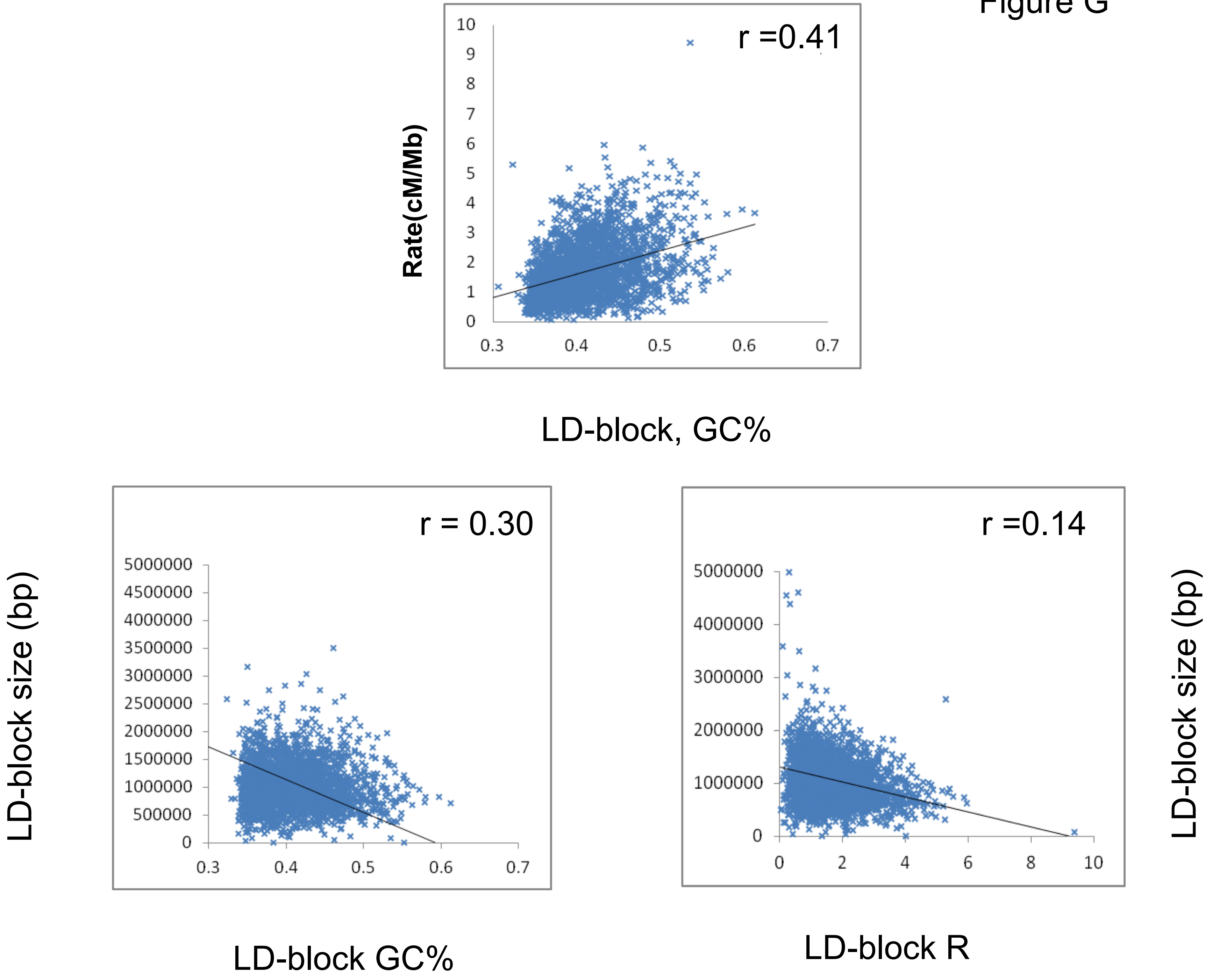
r =0.41
Rate(cM/Mb)
10
9
8
7
6
5
4
3
2
1
0
0.3
0.4
0.5
0.6
0.7
LD-block, GC%
r = 0.30
LD-block size (bp)
5000000
4500000
4000000
3500000
3000000
2500000
2000000
1500000
1000000
500000
0
0.3
0.4
0.5
0.6
0.7
LD-block GC%
r =0.14
LD-block size (bp)
5000000
4000000
3000000
2000000
1000000
0
0
2
4
6
8
10
LD-block R

Figure H

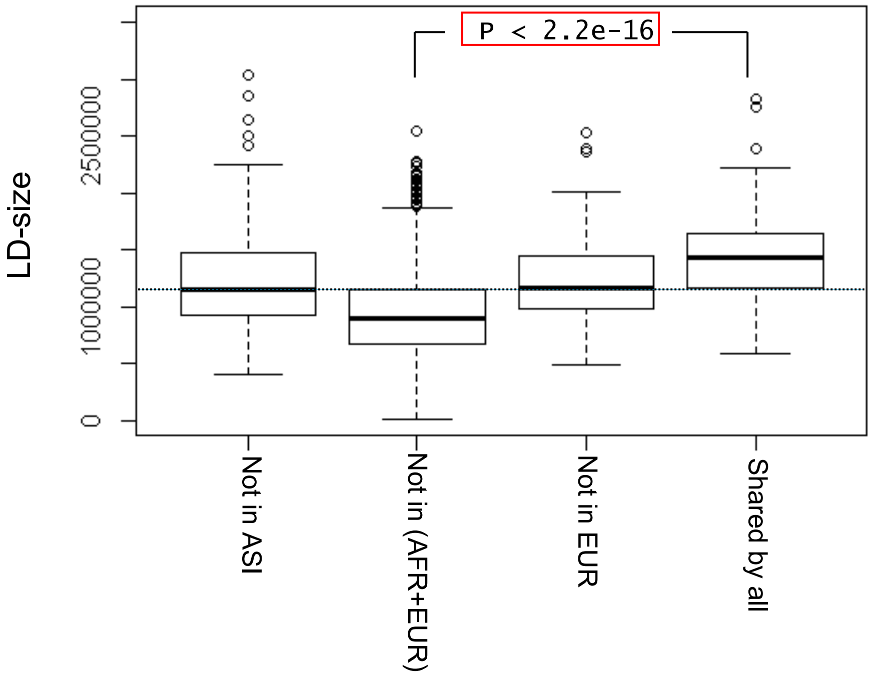

P < 2.2e-16
LD-size
0
1000000
2500000
Not in ASI
Not in (AFR+EUR)
Not in EUR
Shared by all
